# The Beneficial Effects of *Actinomycetales* Immune Modulators in the Pancreas of Diabetic Rats

**DOI:** 10.34172/apb.2021.035

**Published:** 2020-04-22

**Authors:** Monireh Khordadmehr, Solin Ghaderi, Mehran Mesgari-Abbasi, Farinaz Jigari-Asl, Katayoon Nofouzi, Hossein Tayefi-Nasrabadi, Graham McIntyre

**Affiliations:** ^1^Department of Pathobiology, Faculty of Veterinary Medicine, University of Tabriz, Tabriz, Iran.; ^2^Drug Applied Research Center, Tabriz University of Medical Sciences, Tabriz, Iran.; ^3^Department of Basic Sciences, Faculty of Veterinary Medicine, University of Tabriz, Iran.; ^4^Center for Infectious Diseases and International Health, Windeyer Institute for Medical Sciences, University College London, UK.

**Keywords:** Diabetes mellitus, Biochemical indicators, Inflammatory cytokines, Immunotherapy, Histopathology

## Abstract

***Purpose:*** Type 1 diabetes mellitus (T1DM) has dramatically increased in recent years, especially in young people, and limits the life quality of the patients involved. Thus, many researchers are performing extensive studies to find alternative treatments for DM.

***Methods:*** Here, we evaluated the improvement effects of the heat-killed *Actinomycetales* species, including *Gordonia bronchialis*, and *Tsukamurella inchonensis* in streptozotocin (STZ)- diabetic rats by biochemical, immunological, and histopathological examinations.

***Results:*** The present findings exhibited a dramatic and progressive alteration in the serum levels of interleukin-6 (IL-6), IL-10 and tumor necrosis factor-α (TNF-α) in the diabetic group, which were related to the blood glucose and insulin levels, oxidative stress defense (evaluated by TAC and MDA activities), and the pancreas biochemical indicators (such as amylase and lipase). More importantly, the present results were consistent with the histopathological findings, which included cellular degeneration, vascular congestion, hemorrhage, focal necrosis associated with mononuclear cell infiltration. Interestingly, all of the diabetic changes in the blood serum and tissues improved remarkably in the treated groups by *Actinomycetales* species.

***Conclusion:*** Surprisingly, most of the current diabetic complications effectively attenuated after oral administration of both *Actinomycetales* species, particularly with a high dose of *T. inchonensis.* Thus, it is concluded that the heat-killed *Actinomycetales* species can prevent and improve the progression of T1DM and its various complications profoundly.

## Introduction

Type 1 diabetes mellitus (T1DM) is considered as a multifactorial autoimmune disease in which the islet cell immune response destroys insulin-producing β cells in the endocrine islets of Langerhans. The incidence of T1DM has significantly increased in recent years, particularly among young people, which can affect the patient’s life quality. Today, it is well known the contribution of immune-mediated inflammatory mechanisms in the pathophysiology of DM and its complications such as nephropathy and hepatopathy. In this regard, increasing evidence indicated that inflammatory cytokines present a pivotal role in the initiation and progression of T1DM.^1^

Recently, some reports described some aerobic *Actinomycetales* species closely related to mycobacteria including *Rhodococcus coprophilous*, *Gordonia bronchialis*, and *Tsukamurella inchonensis* which are noticeable because of immunomodulatory activi­ties, particularly in heat-killed form.^2^ Growing evidence suggested that both obesity and T2DM can be improved by administration of *Actinomycetales* as an immune modulator.^3^ Up to now, there are no data on the effects of these immune modulators on the pancreatic islets alterations associated with biochemical indicators and inflammatory cytokine profiling in the setting of T1DM. A previous document clarified the critical role of interleukin-6 (IL-6) and tumor necrosis factor-α (TNF-α) in diabetic complications.^4,5^ Thus, the current study evaluated the probable improvement impact of *G. bronchialis* and *T. inchonensis* (as the heat-killed *Actinomycetales* species) on an improvement to pancreatic islet cell disorders in T1DM through histopathological, immunological and biochemical studies.

## Materials and Methods

### Experimental design

Sixty healthy adult male Wistar rats weighing approximately 245–365 g, were purchased and divided randomly into six groups (Table 1). T1DM was induced by an intraperitoneal (i.p) injection of streptozotocin (STZ) (Sigma-Aldrich Co., USA) with 55 mg/kg dosage in five groups. Blood glucose levels were tested three days later (the animals with the blood glucose greater than 250 mg/dL were considered diabetic), the time-point when treatments were initiated. The treatments were conducted according to Table 1 by two different doses (low dose and high dose) of two the heat-killed *Actinomycetales* species including *G. bronchialis* (Gb) and *T. inchonensis* (Ti) (BioEos Ltd, Kent, UK), and also normal saline (for the diabetic and healthy control groups),^3^ which carried out orally for 14 days (consecutively) via stomach gavage needle. The animals were visited every day of the week for 21 days. Blood specimens were obtained after anesthesia (by i.p administration of 50 and 8 mg/kg BW of ketamine and xylazine, respectively) on the 7^th^, 14^th^, and 21^st^ days and sera were discreet at 750 × g for 15 min for subsequent biochemical and immunological tests. Besides, five rats in each group were euthanized, and tissue specimens from the pancreas were collected for histopathological evaluation, which placed and fixed at 10% buffered formalin.

**Table 1:** Different treatments were conducted in the present study (six groups, n = 10)

**Groups**	**Treatment for 14-contineous days**
Low dose Gb	Diabetes treated with 10^5^CFU/rat^*^ *G. bronchialis*
High dose Gb	Diabetes treated with 10^7^CFU/rat *G. bronchialis*
Low dose Ti	Diabetes treated with 10^5^CFU/rat *T. inchonensis*
High dose Ti	Diabetes treated with 10^7^CFU/rat *T. inchonensis*
Diabetic control	Diabetes treated with normal saline
Healthy control	No diabetes treated with normal saline

*CFU/rat: colony forming unit.

### Biochemical assays

#### Serum biochemical indicators evaluation

All of the examined biochemical indicators, including blood glucose levels and serum insulin values, and the activity of amylase and lipase enzymes were measured on the 7^th^, 14^th^ and 21^st^ sampling days by available commercial kits according to the manufacture ʼ s instructions (Pars Azmoon, Tehran, Iran) and using a spectrophotometer (Photometer 5010, Berlin, Germany).

### Serum antioxidant system evaluation

The assays for total antioxidant (TAC) activity and MDA (malondialdehyde) levels were also performed in the obtained sera on the 7^th^, 14^th^, and 21^st^ sampling days using the commercial kits according to the manufacture ʼ s protocol (Pars Azmoon, Tehran, Iran) by a spectrophotometer at 593 and 532 nm, respectively.^6^

### TNF-α, IL-6, IL-10 measurement 

The levels of IL-6, IL-10, and TNF-αwere measured in the stored serum sampleson the 14^th^ and 21^st^ sampling daysusing Rat ELISA commercial kits (Koma Biotech, Korea) according to manufacturerʼs instructions.^7^

### Histopathological examination

The formalin-fixed tissue samples were processed routinely, sectioned, stained firstly with hematoxylin and eosin (H&E), and then Gomori’s Aldehyde Fuchsin method was performed, which finally studied microscopically using a light microscope. The tissue sections were examined for the presence of the pathological changes, including atrophy, necrosis, hemorrhage, and vascular congestion. Moreover, insular area (IA) was evaluated in the pancreas sections according to the following formula that presented by the Committee on Care and Use of Laboratory Animal Resources:

$$AI=\frac{\sum of\;small\;islets\;\left(\sum of\;large\;islets\right)}{\sum of\;observed\;fields\;at\;\times 100\;magnification}$$

Besides, the numbers of β and α cells were counted in tissue sections stained with Gomori’s Aldehyde Fuchsin method, and the portion of β cells to α cells (β/α cells) was recorded.

### Statistical analysis

The present data were analyzed using SPSS software (SPSS, version 16 for windows, USA). As more details, the ANOVA and non-parametric tests were used for statistical analysis of serum parameters and pathological lesions among the various groups, respectively, and a *P* < 0.05 were considered significant.

## Results

### The amylase and lipase levels were significantly improved in the diabetic-treated groups

Lower serum insulin levels associated with higher glucose values were identified in the diabetic-control rats compared to the other treated groups. Interestingly, the higher levels of insulin to gather with the lower levels of glucose were observed in the treated groups by using both bacteria, especially in the Ti-recipient group, with dose dependent-manner (data not shown).

**Figure 1:**
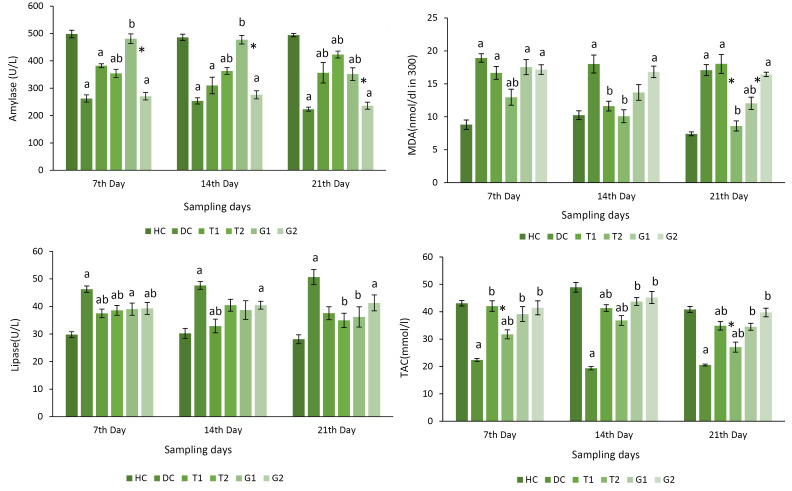
The effects of oral administration of *Actinomycetales* species on the serum levels of amylase **(a)**, lipase **(b)**, MDA **(c)**, and TAC **(d)** on STZ-induced diabetes. Data are presented as the mean ± SD. Differences were considered significant with *P* < 0.05. ^a^ A significant difference with healthy control (HC); ^b^ A significant difference with diabetic control (DC); * A significant difference between low dose and high dose treated groups.

The lower activities of amylase, along with higher levels of lipase (Figure 1a, b), were found in the diabetic rats, which were significantly increased in the diabetic-treated groups. Surprisingly, there was a dose-dependent pattern for amylase alteration.

### The heat-killed bacteria can improve the serum oxidative stress indicators 

The malondialdehyde (MDA) levels (particularly with high-dose of Ti) and TAC activities (particularly with a high-dose of Gb) (Figure 1c, d) significantly differed in the diabetic group rather than the diabetic-treated groups.

### Inflammatory cytokines findings were significantly improved in the heat-killed bacteria recipient groups

**Figure 2:**
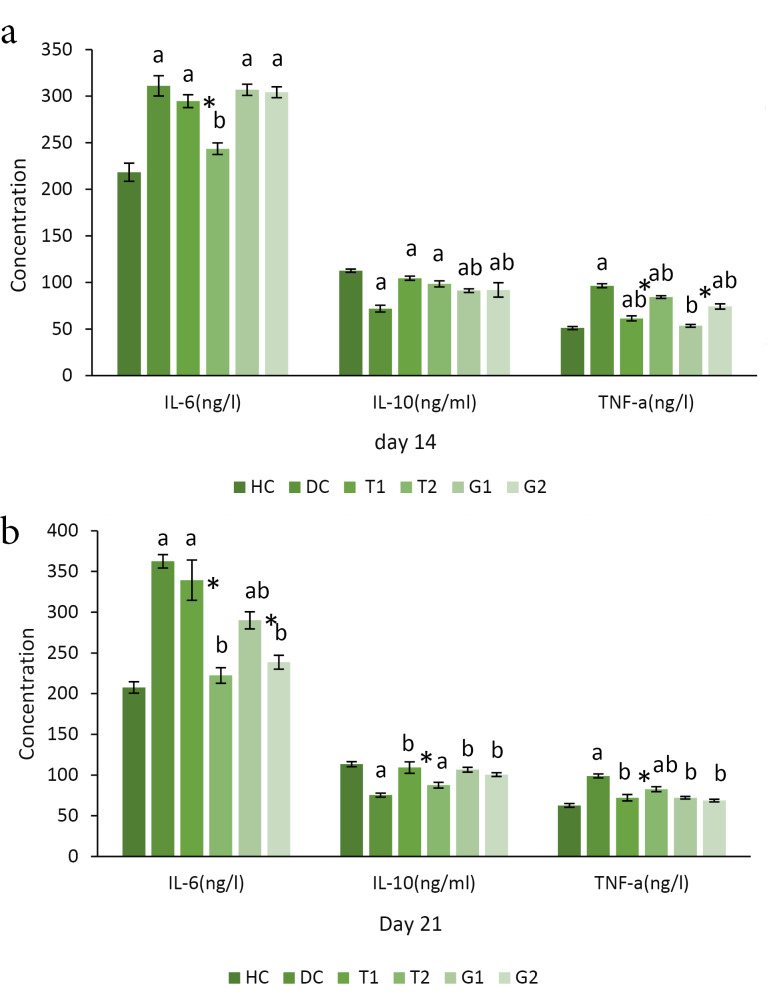
The effects of oral administration of *Actinomycetales species* on the serum levels of IL-6, IL-10, and TNF-α on the 14^th^ **(a)** and 21^st^ (**b)** sampling days on STZ-induced diabetes. Data are presented as the mean ± SD. Differences were considered significant with *P* < 0.05. ^a^ A significant difference with healthy control (HC); ^b^ A significant difference with diabetic control (DC); * A considerable difference between low dose and high dose treated groups.

As expected, we found the notable higher levels of IL-6 and TNF-α pro-inflammatory cytokines together with the markedly lower levels of IL-10 anti-inflammatory cytokines in the diabetic rats as compared to healthy animals, which improved in all diabetic-treated groups (Figure 2a, b). Among these, IL-10 values did not differ between the low dose and high dose groups. By contrast, the values of TNF-α and IL-6 presented significant differences in a dose-dependent manner.

#### Histopathological lesions remarkably reduced in the heat-killed bacteria recipient groups

**Figure 3:**
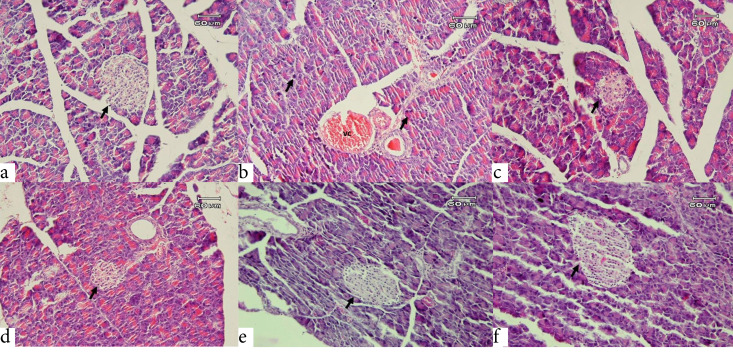
Pancreas, rat, STZ-induced diabetes. (**a)** healthy control with well-defined acini structures and normal islets of Langerhans (arrow). (**b)** diabetic control with degenerative and necrotic changes in both exocrine (arrows) and endocrine parts associated with vacuolar degeneration and vascular congestion (vc). (**c)** low dose Gb-recipient group (G1) with mild improvement of islets of Langerhans (arrow); (**d)** high dose Gb- recipient group (G2) with mild improvement of islets of Langerhans area (arrow); (**e)** low dose Ti- recipient group (T1) with moderate improvement of islets of Langerhans area (arrow); (**f)** high dose Ti-recipient group (T2) with a remarkable improvement of islets of Langerhans (arrow) (H&E).

**Figure 4:**
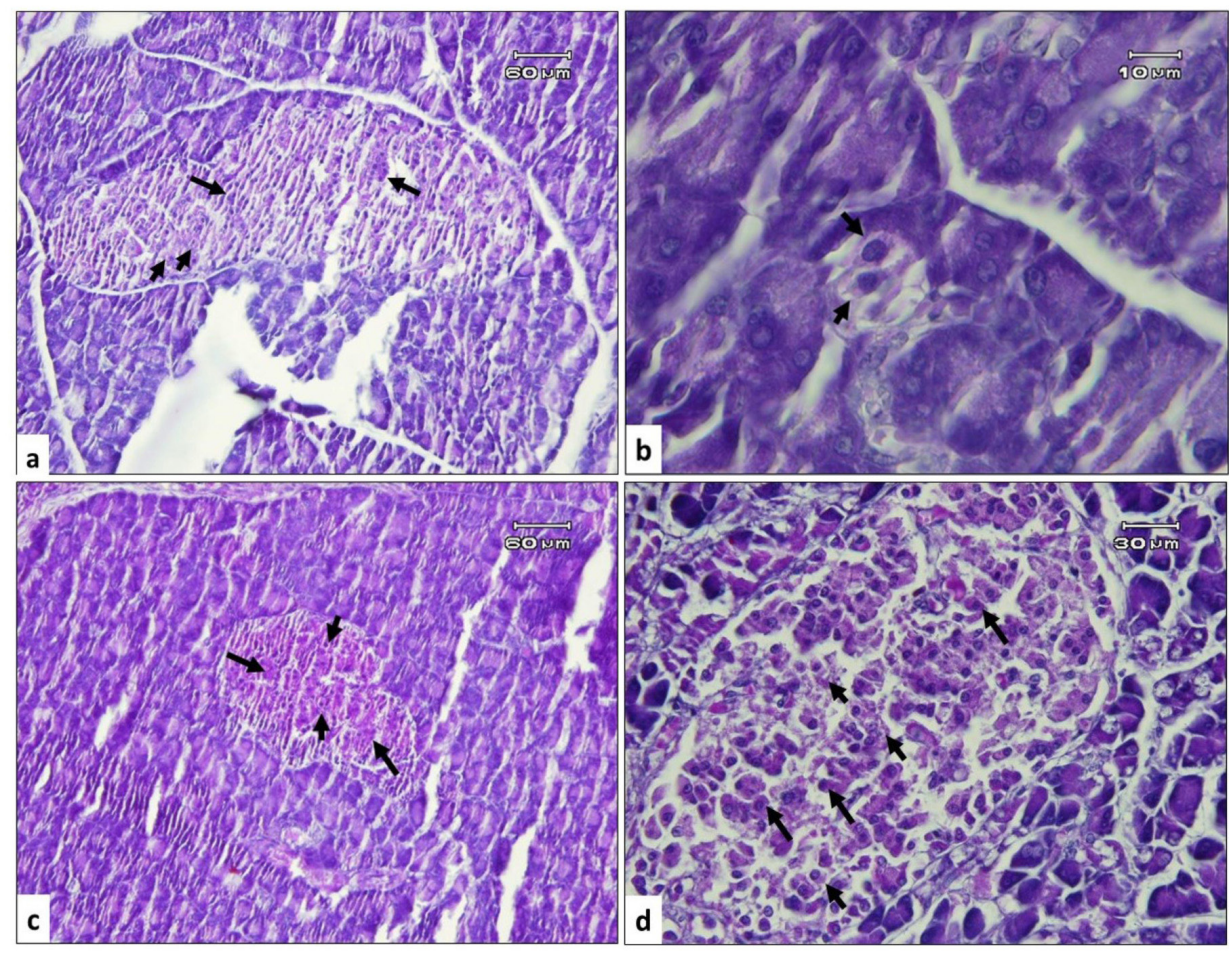
Pancreas, rat, STZ-induced diabetes. short arrows: α-cells; long arrows: β-cells **a:** healthy control with normal β-cells/α-cells in islets of Langerhans; **b:** diabetic control with very small islets of Langerhans and no β-cells; **c:** high dose Gb-recipient group (G2) with moderate improvement of β-cells/α-cells in islets of Langerhans; **d:** high dose Ti-recipient group (T2) with strongly improvement β-cells/α-cells in islets of Langerhans. Gomori’s Aldehyde Fuchsin stain.

Microscopic examination of the pancreas (Figure 3) in the healthy rats demonstrated well-defined acini structures with normal islets of Langerhans. The diabetic animals exhibited degenerative and necrotic changes in both exocrine and endocrine parts, such as the notable disruption and atrophy of Langerhans islets associated with vacuolar degeneration and vascular congestion. Surprisingly, we found strongly significant improvements in the pancreatic structure which clarified by quantitative microscopic evaluation (Table 2), particularly in the size of Langerhans islets (Insular Area) together with β-cells/α-cells (which identified by Gomori’s Aldehyde Fuchsin stain in Figure 4), and at the high dose of Ti in 21^st^ days after treatment which showed nearly normal microscopic morphology. Indeed, there was a progressive degeneration and atrophy of islets in the diabetic control rats from day 14 to 21. In contrast, it was found an incremental improvement in the islets of Langerhans associated with β-cells/α-cells in the treated groups.

**Table 2:** Quantitative microscopic analysis of the Langerhans islets of the pancreas in various experimental groups in the 21 day-post-treatment

**Groups**	**No. small islets** **(** ***P*** ** value=0.12)**	**No. large islets** **(** ***P*** ** value=0.05)**	**Insular Area** **(** ***P*** ** value=0.01)**	**β-cells/α-cells** **(** ***P*** ** value=0)**
HC	1.31 ± 0.39	2.14 ± 0.69	30.29 ± 9.85	3.47 ± 0.24
DC	0.32± 0.11	0.28± 0.14	10.32 ± 3.19	1.12 ± 0.28
T1	0.54±0.19	0.51±0.15	17.26±8.43	2.24±0.16
T2	0.94±0.16	1.86±0.39	28.47±9.38	3.17±0.14
G1	0.62±0.21	0.49±0.17	14.22±5.94	1.96±0.25
G2	0.81±17	1.07±0.28	21.49±7.36	2.52±0.23

HC: healthy control; DC:diabetic control; T1: diabetic rat treated with the low dose of Ti (10^5^CFU/rat); T2: diabetic rat treated with the high dose of Ti (10^7^CFU/rat); G1: diabetic rat treated with the low dose of Gb (10^5^CFU/rat); G2: diabetic rat treated with the high dose of Gb (10^7^CFU/rat).

## Discussion

The present findings exhibited the dramatic and progressive alterations in the serum levels of IL-6, IL-10 and TNF-α in the diabetic group, which were related to the blood glucose and insulin levels, oxidative stress defense (evaluated by TAC and MDA activities), the pancreas biochemical indicators (studied by amylase and lipase). More importantly, these data are in agreement with the present histopathological findings, which included severe pancreatic islet destruction. Accordingly, our results are consistent with other observations in the diabetic animal models.^8,9^ Surprisingly, most of the current diabetic complications induced by STZ were significantly attenuated after oral administration of both *Actinomycetales* species, particularly with a high dose of *T. inchonensis*.

It has been believed that STZ-induced diabetes mellitus and insulin deficiency lead to raised blood glucose and the relation between specific diabetic complications and disturbances in several tissues.^10^ Here, the serum concentrations of glucose and insulin improved with both treatments of Ti and Gb.Similarly, the present observations are constantly with those of some researchers who investigated the noticeable reduction in serum glucose and insulin levels by the same treatment in experimental T2DM and obesity in rats, particularly in the Ti-recipient group.^3^ It was proposed that both obesity and T2DM associated with some major biochemical indicators and kidney-liver tissue damage, can be improved by the administration of *Actinomycetales* as an immune modulator.^3^ In this regard, it seems that interactions of inflammatory mediators involved in DM, the population of inflammatory cells and defense antioxidant system may play an important role in the improvement of pathological lesions.^2^

Growing evidence demonstrated that the pancreatic exocrine activity is affected by the pancreatic endocrine hormones, particularly insulin, which has a trophic impact on the exocrine cells.^11^ Another previous study presented the effect of hyperglycemia on the pancreatic exocrine functions, which significantly modulated with the regulation of glycemic control.^12^ Overall, it was believed that in diabetes mellitus, hyperglycemia, and insulin disorders might lead to the pancreatic exocrine dysfunction and the progression of pancreatic exocrine inadequacy.^13^ Our results revealed the severe progressive destruction of pancreas structures (mainly islets of the pancreas) by STZ administration in the animals.

More importantly, treatment with both *Actinomycetales* species, particularly high dose of *T. inchonensis*, significantly reversed the pancreas structure associated with endocrine (mainly insulin) and exocrine (amylase and lipase) secretions. Convincing evidence postulated that islet (endocrine) hormones could manage pancreatic exocrine activity. Many studies on STZ-induced diabetes in the animals have indicated a decrement in pancreatic enzyme levels,^14^ which were attributed to the insulin reduction caused by β-cell damage, which was reversible upon insulin administration. After injection of STZ, pancreatic exocrine secretion alters, so that, on the 7^th^ day, the higher serum level of lipase along with the lower level of amylase was found. Previous reports published alteration of serum pancreatic amylase and lipase enzymes in patients with T1DM and T2DM.^15,16^ In our experiment, the serum levels of amylase and lipase in the treated groups dramatically modulated compared to the diabetic control group, which is in agreement with the previous studies. In summary, it seems that *Actinomycetales* species can have an improvement effect for attenuation of side effects in various diabetes complications.

It is clarified that oxidative stress represents a critical function in the pathogenesis and development of DM, as well as its related complications. In the present study, TAC activity and MDA levels decreased and increased, respectively, in the diabetic rats with no treatment, which changed effectively by oral administration of both *T. inchonensis* and *G. bronchialis*, particularly in a dose-dependent manner. Indeed, oxidative stress develops in DM through enhanced production of reactive oxygen species, which attribute to excessive lipid peroxidation due to the lipid degradation. Subsequently, it can lead to harmful effects via the accumulation of free radicals, which impairs cell membrane function, leading to the tissue damage.^17^

Here, we observed a marked alteration of the major pro- and anti-inflammatory cytokines involved in diabetes pathogenesis, including IL-10, IL-6, and TNF-α in the diabetic rats, which significantly attenuated after treatment by heat-killed *Actinomycetales* species. As more details, our findings exhibited that alterations of IL-6 and TNF-α were more dose and time-dependent than IL-10. What ҆ s more, it seems that a high dose of Ti can severely manage serum cytokine profile in DM. The inflammatory cytokines originated from peripheral blood T cells, or macrophages provide a crucial role in the induction and progression of diabetes.^18^ Recently, it was proposed that circulating cytokines in DM links with inhibiting the cytotoxicity in pancreas β-cells.^19^ A previous study exhibited that hyperglycemia accelerates the development of the chronic inflammatory processes in patients with DM1.^20^ Accordingly, high levels of glucose promotes IL-6 secretion, which can be described that persistent hyperglycemia provides the formation of advanced glycation end-products.^21^ Additionally, IL-6 modulates insulin secretion. Indeed, low content of IL-6 can promote insulin production, while its high concentration inhibits insulin secretion.^22^

TNF-α is responsible for the progression of T1DM and T2DM through the prevention of glucose-induced insulin secretion and the destruction of β-cell function and inducing apoptosis.^23^ It was pointed that abnormal conditions like hyperglycemia and enhanced oxidative stress can stimulate the activation of the protein complex of the JNK (c-Jun N-terminal kinase) pathway and the NF-κB (nuclear factor Kapa B) signaling, which can release the pro-inflammatory mediators such as IL-6 and TNF-α.^24,25^

It is demonstrated that anti-inflammatory cytokines such as IL-10 (secreted from Th2 cells) exhibited a protective impact against diabetes progression in the animal model.^26^ It was proposed that IL-10 decreased in T2DM, which is associated with risk of DM.^27,28^ In this regard, it is established that it can modulate or inhibit the production of TNF-α and IL-6.^29^ It was also mentioned that the high IL-10 concentrations inhibit the progression of T2DM and its metabolic syndrome by reducing the results of the inflammatory response such as TNF-α and IL-6.^30^

## Conclusion

In conclusion, the present findings indicated that heat-killed *Actinomycetales* species could protect the pancreas functions efficiently in T1DM, which was evidenced by the biochemical indicators, inflammatory cytokine profiles, and the histopathological examination. Overall, it was concluded that oral administration of heat-killed *Actinomycetes* species in the diabetic rats could provide them a potentially strong candidate for industrial application as pharmacological agents for the treatment of some diabetic dysfunctions.

## Ethical Issues

The experiment was authorized by the Research Ethics Committee, Tabriz University of Medical Sciences, Iran (ethical approval code: 5-4-1171, date: 4 May 2013).

## Conflict of Interest

There is no conflict of interest.

## Acknowledgments

The authors express their gratitude to Professor Graham McIntyre, of BioEos Ltd. for the preparation and provision of heat-killed *Actinomycetales* species. This work was supported by the Faculty of Veterinary Medicine, University of Tabriz, Tabriz, Iran, and also the Drug Applied Research Center, Tabriz University of Medical Sciences, Tabriz, Iran.
